# Genetic and functional evidence for gp130/IL6ST-induced transient receptor potential ankyrin 1 upregulation in uninjured but not injured neurons in a mouse model of neuropathic pain

**DOI:** 10.1097/j.pain.0000000000002402

**Published:** 2021-07-08

**Authors:** Theodora Kalpachidou, Philipp Malsch, Yanmei Qi, Norbert Mair, Stephan Geley, Serena Quarta, Kai K. Kummer, Michaela Kress

**Affiliations:** a Institute of Physiology, DPMP, Medical University of Innsbruck, Innsbruck, Austria; b Institute of Pathophysiology, Biocenter, Medical University of Innsbruck, Innsbruck, Austria

**Keywords:** TRPA1, gp130, SNI, Neuropathic pain, DRG, Nerve injury

## Abstract

Peripheral nerve injuries result in pronounced alterations in dorsal root ganglia, which can lead to the development of neuropathic pain. Although the polymodal mechanosensitive transient receptor potential ankyrin 1 (TRPA1) ion channel is emerging as a relevant target for potential analgesic therapies, preclinical studies do not provide unequivocal mechanistic insight into its relevance for neuropathic pain pathogenesis. By using a transgenic mouse model with a conditional depletion of the interleukin-6 (IL-6) signal transducer gp130 in Na_v_1.8 expressing neurons (SNS-gp130^−/−^), we provide a mechanistic regulatory link between IL-6/gp130 and TRPA1 in the spared nerve injury (SNI) model. Spared nerve injury mice developed profound mechanical hypersensitivity as indicated by decreased withdrawal thresholds in the von Frey behavioral test in vivo*,* as well as a significant increase in mechanosensitivity of unmyelinated nociceptive primary afferents in ex vivo skin-nerve recordings. In contrast to wild type and control gp130^fl/fl^ animals, SNS-gp130^−/−^ mice did not develop mechanical hypersensitivity after SNI and exhibited low levels of Trpa1 mRNA in sensory neurons, which were partially restored by adenoviral gp130 re-expression in vitro. Importantly, uninjured but not injured neurons developed increased responsiveness to the TRPA1 agonist cinnamaldehyde, and neurons derived from SNS-gp130^−/−^ mice after SNI were significantly less responsive to cinnamaldehyde. Our study shows for the first time that TRPA1 upregulation is attributed specifically to uninjured neurons in the SNI model, and this depended on the IL-6 signal transducer gp130. We provide a solution to the enigma of TRPA1 regulation after nerve injury and stress its significance as an important target for neuropathic pain disorders.

## Abstract 2

gp130 is a critically important regulator of mechanical hypersensitivity after nerve injury. Transient receptor potential ankyrin 1-responsiveness is upregulated after spared nerve injury in uninjured but not injured neurons. gp130 upregulates transient receptor potential ankyrin 1 in uninjured neurons, and this is associated with signatures of neuropathic pain.

## 1. Introduction

Nerve lesions can lead to the development of neuropathic pain, and pronounced alterations of peripheral and central neurons within the pain pathway have been associated with mechanical hypersensitivity developing after nerve injury in patients and several preclinical models of neuropathic pain.^14,57^ Major neuroinflammatory processes, such as invasion of monocytes and macrophages into the lesioned nerve but also the dorsal root ganglion (DRG), where cell bodies of nociceptive afferents reside—, and the release of inflammatory mediators by immune and local glia cells contribute to the transition from acute towards chronic neuropathic pain.^53,78^ The glycoprotein gp130, which is encoded by IL6ST, acts as the interleukin-6 (IL-6) signal transducing receptor subunit for the entire IL-6 family of cytokines and can be activated by membrane-bound as well as soluble ligand-bound alpha receptor subunits.^31,92^ gp130 homodimerizes only if activated by IL-6/IL-6R complexes and initiates downstream Jak/Stat signaling, which is essential for innate immunity as well as neuronal functions.^31,77^ As a critical regulator of nociceptor sensitivity to heat and punctate mechanical stimuli, IL-6 controls protein synthesis and nociceptive plasticity through convergent signaling to the eIF4F complex.^2,58,65,66,73^ Interleukin-6 signaling acts as a molecular hub that determines neuronal excitability by suppressing the expression of voltage-gated potassium channels.^46^ Ablation of gp130 strongly affects peripheral nerve regeneration but also mechanonociception.^54,72^ The IL-6/IL-6R/gp130 axis is critically involved in neuropathic pain induced by nerve injury, chronic inflammation, cancer, chemotherapy, diabetes, as well as HIV and herpes infections.^39,51,80,99^ Classical as well as trans-signalling cascades have been extensively studied, and therapeutic interventions targeting the IL-6 pathway have been approved mostly for chronic inflammatory disorders.^13,55^ Several treatment strategies have been used targeting IL-6, IL-6R, or gp130 as well as their soluble forms, eg, sgp130Fc that offers promising benefit for diabetic neuropathy and inflammatory disease by specifically blocking IL-6 trans-signaling.^13,55,75,79^

The nonselective cationic channel, transient receptor potential ankyrin subtype 1 (TRPA1 or ANKTM1), has been proposed as a multimodal transducer of noxious cold and mechanical stimuli.^15,16,20,28,32,43,64,84,85^ Increasing pharmacological and genetic evidence moves TRPA1 into focus as a central modifier of pain perception and a very promising drug target of neuropathic pain and mechanical allodynia caused by nerve injury or even diabetes.^1,11,21,26,27,33,36,38,48,59,61,63,70,87,89–91,95^ The mechanism of TRPA1 regulation after inflammation and nerve injury is debated,^8,9,40,44,47,49,56,60,69,71,96^ and relevant roles for TRPA1 and its splice variants have been reported for nociceptors as well as nonneuronal cells, such as glia or immune cells in the peripheral nociceptive system.^17,18,68,81,82,88,100^ Despite the overwhelming evidence supporting the importance of TRPA1 for neuropathic pain and its association with the mechanical allodynia resulting from nerve damage, the mechanisms and signaling cascades regulating TRPA1 expression, in particular in nociceptive primary afferents, are not sufficiently understood. Recent reports link TRPA1 to the proinflammatory cytokine IL-6 in models of chemotherapy-induced and bone cancer pain,^50,97^ and in our previous studies, mice with a conditional deletion of the gp130 in Na_v_1.8 expressing neurons (SNS-gp130^−/−^) exhibit reduced mechanosensitivity associated with decreased levels of TRPA1 in primary nociceptive afferents.^2,54^ Therefore, we set out to address the relevance of the IL6/gp130 to TRPA1 axis for neuropathic pain in the murine model of spared nerve injury (SNI).

## 2. Methods

### 2.1. Transgenic model

SNS-gp130^−/−^ and gp130^fl/fl^ mice were bred and genotyped as previously described.^2^ Unless otherwise stated, adult male mice were used for behaviour phenotyping, whereas mice of either sex and older than 8 weeks were used in all in vitro experiments. Mice were housed under standard pathogen free conditions, at 24°C on a 12:12 light:dark cycle and had ad libitum access to food and water. For tissue dissection, animals were deeply anesthetized with carbon dioxide and euthanized by cervical dislocation. Behavioral measurements and analyses were performed in awake, unrestrained, age-matched, male mice with an age of 8 to 16 weeks by examiners who were blinded to the genotype of the mice. Animals were treated in accordance with ethical guidelines and animal welfare regulations (Medical University of Innsbruck). All experimental procedures were approved by the Austrian National Animal Experiment Ethics Committee of the Austrian Bundesministerium für Wissenschaft und Forschung (BMWF-66.011/0113-II/3b/2010; BMWF-66.011/0051-II/10b/2008).

### 2.2. Spared nerve injury model

**Figure 1.**
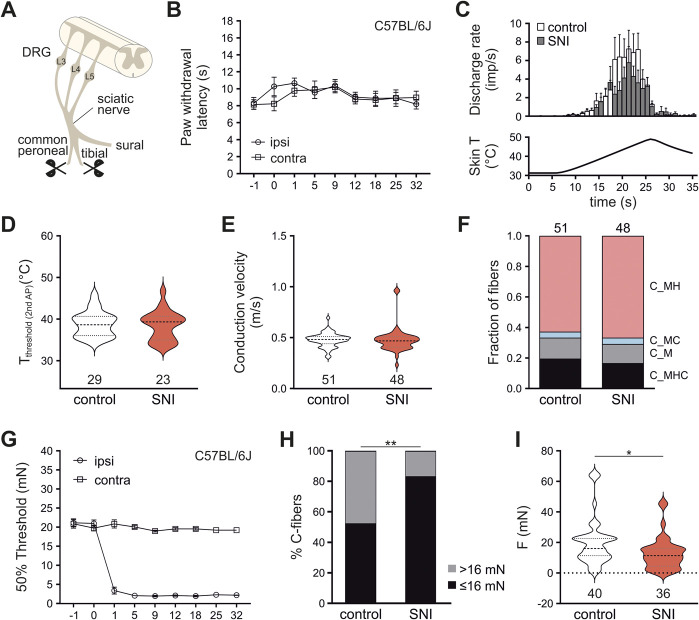
SNI induced mechanical but not heat hypersensitivity in wildtype mice. (A) Schematic representation of SNI. (B–D) Heat sensitivity as indicated by unchanged paw withdrawal latency (PWL) in vivo in the Hargreaves test (N = 8) as well as slightly reduced heat responses and unaltered heat threshold temperatures of polymodal C fibers (SNI: 38.96 ± 0.675°C, n = 23 vs control 39.27 ± 0.523°C, n = 29, *P* = 0.6576, Mann–Whitney *U* test) were not significantly augmented by the SNI treatment. (E) Conduction velocities of C-fibers of the 2 genotypes were not statistically different (Mann Whitney *U* test, *P* = 0.3324). (F) C fibers (C_) were classified as heat (H), cold (C), and mechanical force (M) or combinations and their distribution showed no statistical significance between control and SNI-treated mice (control n = 51 [N = 19]; SNI n = 48 [N = 25]; *P* = 0.9762; χ^2^ = 0.2084). (G) SNI treatment induced a dramatic decrease in mechanical withdrawal thresholds in the von Frey test [2-way repeated measures ANOVA revealed a significant effect of day post-SNI (F_(3.047, 21.33)_ = 74.78, *P* < 0.0001) and injured vs uninjured paw (F_(1.000, 7.000)_ = 7770, *P* < 0.0001) as well as an interaction between the 2 effects (F_(2.173, 15.21)_ = 239.3, *P* < 0.0001), N = 8] that was reflected by corresponding changes in vitro. (H) Mechanical thresholds were significantly altered in nociceptors recorded from SNI-treated animals with a significantly higher number of nociceptors responding to mechanical forces ≤16 mN (Fisher exact test, control: n = 40, SNI: n = 36, *P* = 0.0067). (I) Overall, a significant decrease of mechanical thresholds was observed for nociceptors from SNI mice (SNI median 11.4 mN, n = 36 vs control 16 mN, n = 40; Mann–Whitney *U* test, *P* = 0.0125). *P < 0.05, **P < 0.01. ANOVA, analysis of variance; SNI, spared nerve injury.

The surgery procedure was adopted from Decosterd and Woolf [Fig. 1A].^19^ In brief, under xylazine (0.2 mg/kg, AniMedica, Senden-Bösensell, Germany) and ketamine (2 mg/kg, Graeub, Bern, Switzerland) anesthesia, the skin on the lateral surface of the thigh was incised and the sciatic nerve was exposed by separating the biceps femoris through incision of the connective tissue without wounding the muscle. For the SNI procedure, the common peroneal and the tibial nerve were ligated with 4-0 Vicryl (Sh-1 plus; Ethicon, Vienna, Austria) and a portion of 3 mm length was excised around the ligation site. Care was taken to avoid any mechanical damage to the sural nerve. After dissection, muscle and skin were sutured using 4-0 Vicryl. Sham treatment involved exposure of the sciatic nerve without ligation and dissection of the nerves. Mice were left to recover at 37°C until they regained consciousness.

### 2.3. Retrograde neuronal labeling

Sensory neurons were labeled with either dextran or DiI/DiO. Two µL of Texas Red-labeled 3000 Da dextran tracer (5% in saline, Invitrogen, ThermoFisher Scientific, Waltham, MA) were injected 7 days before SNI in the center of the hind paw plantar to retrograde label DRG from the injured tibial or common peroneal nerve or in the lateral hind paw plantar area immediately after the SNI surgery to retrograde label DRG from the noninjured sural nerve. For DiI/DiO, sensory neurons projecting their afferent fibers to the hind paw were retrogradely traced by intracutaneous injection of 10 µL 1 mg/mL DiI (D282/DiIC_18_(3), Molecular Probes, Vienna, Austria) in 4% DMSO (Sigma, Vienna, Austria) in PBS (PAA, Vienna, Austria) into the lateral, plantar side of the hind paw under brief isoflurane anesthesia. Alternatively, DiI or in some cases DiO crystals were deposited near the cut nerve stumps of the saphenous and anterior tibial nerves right after nerve transection 7-28 days before DRG neurons were used in experiments.

### 2.4. von Frey and Hargreaves sensory testing

Sensitivity to heat and mechanical stimuli were assessed twice before surgery (baseline measurements days −1 and day 0) as well as on the indicated days after SNI or sham operation on both paws in a blinded manner, as previously described.^2,72,73^ In brief, for mechanical sensitivity, calibrated von Frey filaments (2.8, 4, 5.7, 8, 11.4, 16, 22.6, 32, and 45.3 mN) were applied to the lateral side of the plantar surface of the paw (sural nerve innervation territory) and the withdrawal threshold was calculated according to the up-and-down method.^12,23^ For assessment of heat hypersensitivity, animals were placed into a Perspex box with a transparent glass floor (Hugo Basile). Heat stimuli were applied to the plantar surface of the hind paws of the mice by the Hargreaves apparatus,^35^ which uses focused infrared light onto the hind paw. The light automatically cut off when the paw was removed or 20 seconds after it was switched on, and the latency at shut off was recorded as paw withdrawal latency.

### 2.5. Skin-nerve preparation and single fiber recordings

Sham- or SNI-treated wild type (wt) mice were used to obtain skin-nerve preparations, and standard single-fiber recordings were performed as we described previously.^45^ In brief, the sural nerve and innervated skin of the hind paw were dissected; the preparation was placed corium side up in an organ bath chamber and superfused (appr. 12 mL/minute) with an oxygen-saturated modified synthetic interstitial fluid containing (in mM) 108 NaCl, 3.48 KCl, 3.5 MgSO_4_, 26 NaHCO_3_, 1.7 NaH_2_PO_4_, 2.0 CaCl_2_, 9.6 sodium gluconate, 5.5 glucose, and 7.6 sucrose at a temperature of 31.5 ± 0.8°C and a pH of 7.4 ± 0.05. The distal end of the sural nerve was pulled into a separate chamber and electrically isolated from the bath solution using paraffin oil. Fine nerve strands dissected from the nerve bundle were placed on a gold wire recording electrode. Action potentials were recorded, amplified (up to 5000-fold), filtered (low pass 1 KHz, high pass 100 Hz), visualized, and stored or analyzed on a PC-type computer with the Spike/ Spidi software package.^29^ The receptive field was first identified, and activation threshold and conduction velocities of nerve fibers were determined. The fibers were characterized as unmyelinated (C) according to their conduction velocity (<1.0 ms^−1^). The mechanical threshold of each unit was determined with calibrated von Frey filaments with a uniform tip diameter of 0.8 mm by applying increasing forces from 1 mN to up to 256 mN, starting with a filament of 22.6 mN. A feedback controlled radiant heat source was used for standard heat stimulation linearly increased the intracutaneous temperature at the receptive field from 31 to 50°C within 20 seconds. For cold stimulation, ice cold synthetic interstitial fluid was applied to a metal ring isolating the receptive field from the bath which decreased the temperature from 31 to 3°C within 4 seconds and held this temperature for 20 seconds. Fibers were considered sensitive if 5 or more action potentials were evoked during the stimulus. The threshold was defined as the force or temperature that elicited the second spike of the response.

### 2.6. Dorsal root ganglia neuron culture and microfluorimetric calcium measurements

After SNI, lumbar dorsal root ganglia L3 to L5 with the cell bodies of primary afferents that project into the lesion were harvested, treated enzymatically, and dissociated as we described previously.^54^ The resulting cell suspension was plated on coverslips coated with poly-L-lysin and laminin and cultivated in serum-free and defined medium (TNB-100 basal medium) medium (Biochrom) supplemented with nerve growth factor (NGF 25 ng/mL), L-glutamine, penicillin G sodium, and streptomycin sulfate (all from Invitrogen) at 37°C in 5% CO_2_. Microfluorimetric Ca^2+^ measurements were performed as previously described.^10^ After 2 to 24 hours, the cultures after nondisruptive loading with 3 or 6 µM of the Ca^2+^ sensitive dye Fura-2 AM (Invitrogen) were recorded in extracellular solution containing (in mM): 145 NaCl, 5 KCl, 2 CaCl_2_, 1 MgCl_2_, 10 D-glucose (all from Sigma), and 10 HEPES (Roth, Karlsruhe, Germany), at pH 7.3 adjusted with NaOH (Merck). Ratiometric measurements were performed using a Zeiss Axiovert 200 microscope (Zeiss) with a Fluar 20x/0.75 N.A. objective (Zeiss). Fura-2 was excited consecutively at 340 and 380 nm (equal excitation time 55 ms) with a polychrome IV monochromator (TILL Photonics, Gräfelfing, Germany). Fluorescence was filtered by a 510 nm long pass filter and recorded with a CCD camera (CoolSNAP, Roper Scientific, Munich, Germany) using 8 × 8 binning at 1 second intervals. For data acquisition, MetaFluor 7.1.2.0 (Molecular Devices, Biberach an der Riss, Germany) was used, data traces were filtered by a simple 3-point moving average and off-line analysis was performed with Excel 2007 (Microsoft). All chemicals were diluted in extracellular solution and applied by a gravity driven perfusion system.^22^ Only cells with a low and stable baseline Ca^2+^ ratio (<1) were used for analysis. The inclusion criterion for cells responsive to cinnamaldehyde (CA) or capsaicin (Caps) was set to 125% percent increase above baseline ratio 10 seconds before stimulation. Viability of each neuron was tested with a 10 seconds pulse of 25 mM potassium chloride solution. All chemicals were purchased from Sigma. Retrograde labeling was visualized using a 549-nm excitation wavelength with a matching filter set (band pass filter BP 575-640, Zeiss), a Fluar 20x/0.75 N.A. objective (Zeiss), 2 × 2 binning, and 100 ms exposure time.

### 2.7. mRNA quantification

RNA was extracted from lumbar L3-5 DRG explants of gp130^fl/fl^ and SNS-gp130^−/−^ mice subjected to the sham or SNI surgery. PeqGOLD TriFast reagent (Peqlab Biotechnologie, Germany) was used in accordance to manufacturer's instructions [chloroform (C2432) and absolute ethanol (107017) were obtained from Merck]. The RNA pellet was diluted in nuclease free water (R0582, ThermoFisher Scientific), and RNA concentration was estimated using NanoDrop 2000 (ThermoFisher Scientific). Reverse transcription of total mRNA was performed as previously described.^46^ Genes of interest were quantified by reverse transcription quantitative polymerase chain reaction using TaqMan Gene Expression Assays: Trpa1 (Mm00625268_m1), Trpv1 (Mm01246302_m1), Piezo1 (Mm01241549_m1), Piezo2 (Mm01265861_m1), Hprt (Mm00446968_m1), Sdha (Mm01352363_m1), and Tfrc (Mm00441941_m1). Hprt, Sdha, and Tfrc were used as reference genes. Reactions were prepared according to manufacturer's instructions and loaded on MicroAmp Fast Optical 96-well reaction plates for amplification in duplicates alongside nontemplate controls. (7500 Fast RT-PCR system, ThermoFisher Scientific). The cycling protocol was 10 minutes at 95°C and 40 2-step cycles of 15 seconds at 95°C and 1 minute at 60°C. Threshold was set manually at 0.1, and baselines were automatically calculated. Relative gene expression was calculated using the 2^*−ΔCt*^ method and expressed in relation to the respective expression of the geometric mean of the 3 reference genes or the 2^*−ΔΔCt*^ method in which case the expression levels were depicted as fold change normalized to the control condition. No signal was detected in the nontemplate controls.

### 2.8. Adenovirus vector construction and dorsal root ganglion cultures transduction

Plasmids for the gp130 adenoviral construct were produced according to the Gateway system procedure (Invitrogen).

#### 2.8.1. Polymerase chain reaction products

For a first polymerase chain reaction (PCR), the primers _a and _c were used to add a Kozak sequence at the 5'end and anchors for *att*B1 and *att*B2 sites at the 5′ and 3′ ends of the gp130 gene. In a second PCR, the *att*B sites were completed using the primers 1107_20 and 1106_20.

Primer_a: *5′* CAAAAAAGCAGGCTCCATGTCAGCACCAAGGATTTGGC.

Primer_c: *5′ CAAGAAAGCTGGGTCCTGCGGCATGTAGCCAC*.

#### 2.8.2. BP-reaction

The BP Clonase enzyme mix was used to recombine the gp130 gene flanked by *att*B sites with the donor vector pDONOR207. The donor vector confers gentamycin resistance and contains the ccdB gene, ie, lethal for E. coli flanked by *attP* sites. In case of successful recombination, the resulting vector was an entry clone in which the gp130 gene is flanked by *att*L sites. Cells were transformed with the plasmids and grown on selective media containing gentamycin. After plasmid isolation, the resulting pENTR vector (pDONOR207-mgp130) was verified by DNA sequencing.

#### 2.8.3. LR reaction

In this step, the gp130 gene flanked by *attL* sites (entry clone) was transferred into a destination vector with *attR* sites using an LR Clonase enzyme mix (Invitrogen). The destination vector contains a dest cassette, which is exchanged by the recombination, the V5 epitope, and an ampicillin resistance gene. The final vector was an expression clone containing the gp130 gene tagged with the V5 epitope: pAd/CMV-mgp130-V5.

#### 2.8.4. Virus production

HEK 293T cells were transfected with the expression clone and grown until confluence. The cells were lysed, and the supernatant was collected for further amplifications. The collected supernatant was purified using Vivapure AdenoPACK 20 kit (Sartorius Stedim Biotech GmbH, Gottingen, Germany; Cat. # VS-AVPQ020).

#### 2.8.5. Dorsal root ganglion neuron cultures transduction

Dorsal root ganglion neurons were transduced with 0.1 to 10 µL/mL pAd/CMV-mgp130-V5 adenovirus and cultured for 48 hours. The concentration of 1 µL/mL of virus was nontoxic and used for this study. An empty pAd/CMV-V5 was used as control.

### 2.9. Statistical analysis

Statistical analysis was performed per animal (*N*) and per nerve fiber or number of cells (*n*). For statistical analysis, Sigma Stat 3, Origin Pro 8, and GraphPad Prism 9 software were used. Depending on sample size, distribution, and number of variables, appropriate statistical tests were used and are indicated in the figure legends. Violin plots indicate median (thick dashed line) and first and third quartile (thin dashed lines) Differences were considered statistically significant at *P* < 0.05.

## 3. Results

### 3.1. Spared nerve injury induced mechanical but not heat hypersensitivity in vivo and ex vivo

As expected, and in line with previous reports,^19,42,93^ SNI-treated mice (Fig. 1A) showed unaltered thermal withdrawal behavior throughout the entire observation period. Heat nociception, as indicated by heat-induced paw withdrawal latency, was similar in both paws in the Hargreaves test in vivo (Fig. 1B). Correspondingly, heat responses and threshold temperatures of unmyelinated heat-responsive C fibers in vitro were comparable with controls (Fig. 1C and D). Likewise, conduction velocities were similar in control and SNI-treated primary nociceptors (Fig. 1E). Analysis of the different C-fiber subpopulations did not indicate loss or gain of specific neuron populations, which suggests that the overall composition of uninjured nociceptive primary afferents within the sural nerve was not affected by SNI (Fig. 1F). In contrast and importantly, SNI induced a severe and persistent decrease in mechanical von Frey thresholds in vivo (Fig. 1G), which was accompanied by a significant increase of the number of mechanosensitive C fibers responding to low mechanical stimuli in the sural nerve: More than 80% of nociceptors responded to mechanical forces below 16 mN after SNI (Fig. 1H). Overall, mechanical thresholds of unmyelinated primary afferents were significantly decreased in the ex vivo skin nerve preparation 7 days after injury (Fig. 1I).

### 3.2. gp130 depletion ameliorated mechanical hypersensitivity

Numerous reports link the proinflammatory cytokine IL-6 and its signal transducer gp130 to mechanical hypersensitivity, and in our previous study, mice with a selective depletion of gp130 in neurons expressing the nociceptor-specific voltage-gated sodium channel Na_v_1.8 show pronounced mechanical hyposensitivity.^54^ Therefore, we hypothesized that gp130 not only determines mechanosensitivity of nociceptors in healthy mice but also could be causally involved in the induction of neuropathic mechanical hypersensitivity.

**Figure 2.**
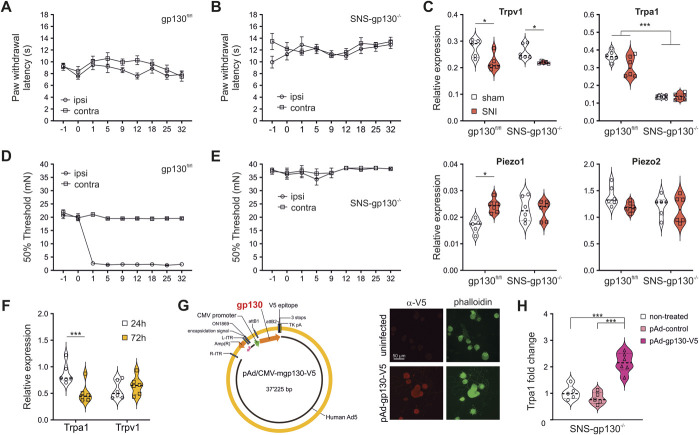
SNI-induced mechanical hypersensitivity requires gp130 expression in Na_v_1.8 expressing primary afferent neurons. (A–B) Like wild type controls, floxed mice as well as mice with a conditional depletion of gp130 from nociceptive neurons do not develop SNI-induced ipsilateral nor contralateral thermal hypersensitivity. (C) Top: SNI did not induce the upregulation of the heat transducer ion channel Trpv1, which was found slightly downregulated after SNI in both genotypes (mean ± SEM for gp130^fl/fl^: sham 0.2748 ± 0.015 and SNI 0.2207 ± 0.014, Welch *t* test t_(7.961)_ = 2.61, *P* = 0.031, N = 5 per group; for SNS-gp130^−/−^: sham 0.2594 ± 0.011 and SNI 0.2199 ± 0.002 Welch *t* test t_(5.33)_ = 3.518, *P* = 0.015, N = 6 for sham and 5 for SNI). The quantification of Tpra1 mRNA expression confirmed the lower expression levels in the DRG obtained from SNS-gp130^−/−^ vs gp130^fl/fl^ mice (mean ± SEM gp130^fl/fl^: 0.3661 ± 0.01185 vs SNS-gp130^−/−^: 0.1356 ± 0.0036, unpaired *t* test t_(10)_ = 18.607, *P* < 0.001, N = 6 per group). SNI did not lead to increased Trpa1 mRNA expression, and this was similar in both genotypes. Bottom: No significant change was observed for the mechanotransducer ion channel Piezo2, whereas Piezo1 increased significantly, but its very low expression levels makes it unlikely that this channel plays a major role in nociceptive mechanotransduction. (D–E) In contrast to wildtype and gp130^fl/fl^ controls [2-way RM ANOVA revealed a significant effect of day post-SNI (F _(1.232, 8.623)_ = 85.21, *P* < 0.0001) and injured vs uninjured paw (F_(1.000, 7.000)_ = 1400, *P* < 0.0001) as well as an interaction between the 2 effects (F_(2.694, 18.86)_ = 201.3, *P* < 0.0001), N = 8], mechanical sensitivity is unaltered in SNS-gp130^−/−^ after SNI. (F) In neuronal DRG cultures derived from gp130^fl/fl^ control mice, Trpa1 mRNA levels decreased after 72 hours (unpaired *t* test t_(10)_ = 3.347, *P* = 0.0074, N = 6), whereas Trpv1 expression remained unchanged. (G) Scheme of the viral construct pAd/CMV-mgp130-V5 used for transduction of DRG neurons and representative photomicrograph of infected primary DRG SNS-gp130^−/−^neuronal cultures (24 hours) stained for V5 tag. (H) Adenoviral re-expression of gp130 increased and partially restored the expression of Trpa1 mRNA in neuronal cultures from SNS-gp130^−/−^ mice (one-way ANOVA F_(2,15)_ = 26.55, *P* < 0.0001, N = 6 per group). *P < 0.05, **P < 0.01, ***P < 0.001. ANOVA, analysis of variance; DRG, dorsal root ganglion; SNI, spared nerve injury; TRPA1, transient receptor potential ankyrin 1.

In SNS-gp130^−/−^ mice, heat sensitivity before and after SNI was unaltered and similar to their control gp130^fl/fl^ littermates (Figs. 2A and B). This was further associated with similar expression of the heat transducer ion channel TRPV1 in DRG from SNI-treated SNS-gp130^−/−^ vs gp130^fl/fl^ control mice. Trpv1 mRNA levels were not affected by the genotype but appeared to slightly decrease after surgery, which may indicate injury-induced fiber loss, however, is unlikely to contribute to nociceptor sensitization to mechanical stimuli (Fig. 2C top left).

In contrast and in line with our hypothesis, SNS-gp130^−/−^ mice significantly differed from their littermate controls by the complete absence of signs of mechanical hypersensitivity at the treated paw after SNI (Figs. 2D and E). Because gp130 deficient mice are protected from SNI-induced mechanical hypersensitivity and show decreased Trpa1 expression,^54^ we hypothesized that TRPA1 upregulation could be causally involved in SNI-induced nociceptor sensitization to mechanical stimuli. Reverse transcription quantitative polymerase chain reaction confirmed significantly lower expression of TRPA1 mRNA in SNS-gp130^−/−^ DRG, which was, however, unaltered after SNI (Fig. 2C top right). In addition, the mRNA expression levels of 2 other mechanically sensitive ion channels, Piezo1 and Piezo2, were unaffected by nerve injury (Fig. 2C bottom). This indicated that there was no overall upregulation of TRPA1 after nerve injury during the observed time window in acute DRG explants. Interestingly, axotomized cultured primary sensory neurons derived from gp130^fl/fl^ mice downregulated Trpa1, but not Trpv1, in a time-dependent manner, indicating that in culture, axotomized sensory neurons adopt an increasingly neuropathic phenotype that seems to make them a model more suitable to study injured rather than uninjured peripheral neurons (Fig. 2F).^30^ Based on these considerations, we hypothesized that Trpa1 expression was regulated by gp130-dependent signaling pathways and used an adenoviral vector construct to overexpress gp130 in cultured DRG neurons obtained from SNS-gp130^−/−^ and control gp130^fl/fl^ mice (Fig. 2G). After 48 hours of viral gp130 re-expression in DRG neurons derived from SNS-gp130^−/−^ mice, Trpa1 mRNA expression was significantly increased compared with the nontreated and control virus–infected cultures (Fig. 2H).

### 3.3. Reduced responsiveness to cinnamaldehyde in neurons from SNSgp130^−/−^ mice

**Figure 3.**
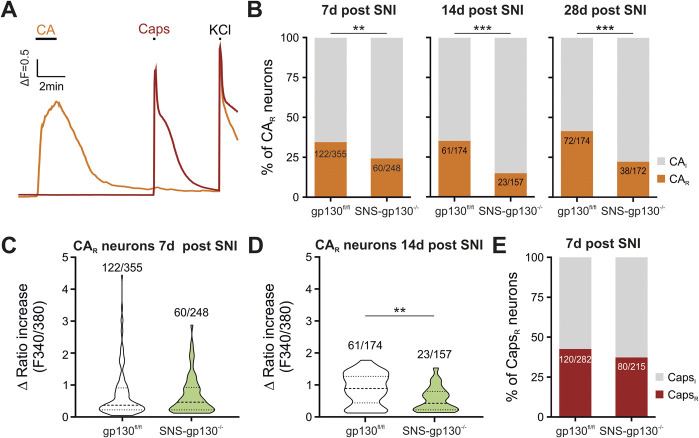
Reduced TRPA1-responsiveness in SNS-gp130^−/−^ neurons after SNI. (A) Representative recording of Ca^2+^ transients responding to 200 µM cinnamaldehyde (CA) and 100 nM capsaicin (Caps). (B) 7 days after SNI, a significantly smaller percentage of small size SNS-gp130^−/−^ neurons responded to CA (CA_R_) in comparison with gp130^fl/fl^ controls (Fisher exact test, gp130^fl/fl^: n = 355; SNS-gp130^−/−^: n = 248, *P* = 0.0089). This difference between the 2 genotypes became even more pronounced at later stages of SNI with less total CA-responsive SNS-gp130^−/−^ neurons at 14 (Fisher exact test, gp130^fl/fl^: n = 174; SNS-gp130^−/−^: n = 157, *P* < 0.0001) and 28 days after SNI (Fisher exact test, gp130^fl/fl^: n = 174; SNS-gp130^−/−^: n = 172, *P* = 0.0001). (C–D) The magnitude of CA evoked Ca^2+^ transients was unaltered 7 days after SNI (gp130^fl/fl^ mean ± SEM: 0.7084 ± 0.0702, n = 122; SNS-gp130^−/−^ mean ± SEM: 0.6682 ± 0.0785, n = 60, *P* = 0.780, Mann–Whitney *U* test) but developed a decrease in SNS-gp130^−/−^ DRG neurons 14 days after SNI, indicating a further decay of TPRA1 at later stages of neuropathy (for gp130^fl/fl^ 0.8789 ± 0.062 and for SNS-gp130^−/−^ 0.5480 ± 0.081, Mann–Whitney *U* test *P* = 0.0054). (E) The percentage of neurons responding to Caps (Caps_R_) was similar in both genotypes (Fisher exact test, gp130^fl/fl^: n = 282; SNS-gp130^−/−^: n = 215, *P* = 0.2320). For 7 days N = 10/group, for 14 days N = 7/group, and for 28 days N = 4/group. CA_R_: cinnamaldehyde responsive neurons, CA_I_: cinnamaldehyde irresponsive neurons, Caps_R_: capsaicin responsive neurons, Caps_I_: capsaicin irresponsive neurons. *P < 0.05, **P < 0.01, ***P < 0.001. DRG, dorsal root ganglion; SNI, spared nerve injury; TRPA1, transient receptor potential ankyrin 1.

As Trpa1 mRNA expression was profoundly compromised in SNS-gp130^−/−^ compared with gp130^fl/fl^ mice independent of sham or SNI treatment, we hypothesized that a posttranslational regulation by gp130 could be a plausible explanation. In line with our previous study, the responsiveness to the TRPA1 agonist cinnamaldehyde (CA) of untreated DRG neurons from SNS-gp130^−/−^ DRG was profoundly reduced as compared with gp130^fl/fl^ mice.^54^ To assess possible functional deficits related to neuropathic pain after injury, we explored the responsiveness of acutely isolated DRG neurons to CA by microfluorimetric intracellular calcium measurements. The differences in TRPA1 mRNA expression were mirrored by a reduced CA responsiveness of acutely isolated neurons obtained from SNI-treated mice in several ways (Fig. 3): 7 days after SNI injury, the percentage of neurons that responded to CA was significantly reduced in SNS-gp130^−/−^ compared with neurons from gp130^fl/fl^ mice 7 days after SNI, and this difference was maintained and even enhanced at 14 and 28 days (Fig. 3B). The overall magnitude of the individual responses to CA did not yet differ between genotypes at 7 days but became significant at 14 days after SNI (Figs. 3C and D). By contrast, responses to the TRPV1 agonist capsaicin were unaltered after SNI (Fig. 3E). These results further support the general dependence of TRPA1 expression in sensory neurons on the presence of gp130.

### 3.4. Spared nerve injury induced upregulation of transient receptor potential ankyrin 1 responsiveness specifically in uninjured neurons expressing gp130

**Figure 4.**
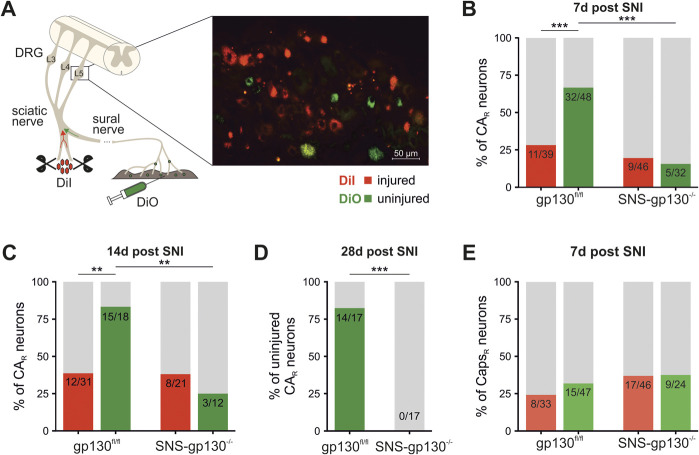
Reduced TRPA1-responsiveness in SNS-gp130^−/−^ uninjured neurons. (A) Schematic representation of DiI/DiO application and representative photomicrograph of a DRG section. DiI and DiO were applied to the nerve stumps and into the innervation territory of the intact sural nerve, as indicated, which separated populations of green and red neurons in the respective lumbar DRG. (B) 7 days after SNI, uninjured neurons from gp130^fl/fl^ exhibited increased CA-responsiveness (Fisher exact test, gp130^fl/fl^ injured: n = 39; gp130^fl/fl^ uninjured: n = 48, *P* = 0.0005), whereas in SNS-gp130^−/−^ uninjured cells, CA responsiveness was significantly diminished (Fisher exact test, gp130^fl/fl^ uninjured: n = 48; SNS-gp130^−/−^ uninjured: n = 32, *P* < 0.0001). (C) The percentage of uninjured CA-responsive neurons remained significantly increased 14 days after SNI in gp130^fl/fl^ controls, compared with injured neurons (Fisher exact test, gp130^fl/fl^ injured: n = 31; gp130^fl/fl^ uninjured: n = 18, *P* = 0.0031) and unaltered between injured and uninjured neurons in SNS-gp130^−/−^ mice (Fisher exact test, SNS-gp130^−/−^ injured: n = 21; SNS-gp130^−/−^ uninjured: n = 12, *P* = 0.7026), indicating a persisting difference in CA-responsiveness of uninjured neurons between the 2 genotypes (Fisher exact test, gp130^fl/fl^ uninjured: n = 18; SNS-gp130^−/−^ uninjured: n = 12, *P* = 0.0024). (D) A further reduction in CA-responsive SNS-gp130^−/−^ uninjured neurons was observed 28 days after SNI (Fisher exact test, gp130^fl/fl^ injured: n = 17; gp130^fl/fl^ uninjured: n = 17, *P* < 0.0001). (E) No differences in Caps responses were observed in injured and uninjured neurons. For 7 days N = 10/group, for 14 days N = 7/group, and for 28 days N = 4/group. CA_R_: cinnamaldehyde responsive neurons, CA_I_: cinnamaldehyde irresponsive neurons, Caps_R_: capsaicin responsive neurons, Caps_I_: capsaicin irresponsive neurons. *P < 0.05, **P < 0.01, ***P < 0.001. DRG, dorsal root ganglion; SNI, spared nerve injury; TRPA1, transient receptor potential ankyrin 1.

Neurons respond to a peripheral nerve injury in a distinct manner depending on whether they are directly injured or indirectly affected by the lesion as injured as well as uninjured axons travel alongside within the same nerve in the SNI model.^4,5^ Based on our observation that TRPA1 expression decreases in axotomized (injured) neurons harvested for primary sensory neuron culture (Fig. 2F), we retrogradely labeled neurons in vivo with Dextran dyes or DiI/DiO (Fig. 4A), to distinguish between injured and uninjured neurons. Most uninjured neurons, but only few injured neurons of control mice, responded to CA 7 days after SNI. In addition, this approach revealed a striking difference between the 2 genotypes with dramatically and specifically increased numbers of CA-responsive uninjured neurons obtained from gp130^fl/fl^ mice but not from SNS-gp130^−/−^ mice (Fig. 4B). The differential regulation of CA responsiveness became even more pronounced 14 and 28 days after SNI when the percentage of uninjured neurons responding to CA further increased to about 80% in control but not in SNSgp130^−/−^ mice after 14 (Figs. 4C) and 28 days (Fig. 4D). By contrast, uninjured neurons obtained from SNS-gp130^−/−^ mice became increasingly insensitive to CA. No comparable changes were obtained for capsaicin sensitivity (Fig. 4E). These findings suggest a very specific gp130-dependent upregulation of TRPA1 in uninjured neurons in the SNI model, which may be occluded by TRPA1 downregulation in injured neurons when analyzing TRPA1 expression in DRG explants. These results provide a solution for the ongoing controversial discussion on TRPA1 expression in painful neuropathies and further stress the critical importance of a gp130-dependent increase of TRPA1 for neuropathic pain.

## 4. Discussion

It is generally accepted that the mechanosensitive ion channel TRPA1 contributes to the development of neuropathic pain and hypersensitivity. Its importance for neuropathic changes emerges not only in nociceptive primary afferents but also in Schwann cells and macrophages.^17,18,62,82^ Therefore, it is not surprising that TRPA1 has been proposed as one of the most promising targets for analgesic drug development.^83^ Despite seminal preclinical studies stressing the importance of TRPA1 for neuropathic pain and the transition towards pain chronification, mechanistic insight into the regulation of the channel and how it contributes to the pathogenesis of neuropathic pain is still largely missing.^88^

The murine SNI model offers the unique advantage to separately explore injured and uninjured neurons lying next to each other within the DRG, which is not the case in other traumatic neuropathy models, such as spinal nerve ligation or chronic constriction injury. We discovered that injured and uninjured neurons regulate TRPA1 expression in an inverse manner in this model: While injured neurons lost responsiveness to TRPA1 agonists within 7 to 14 days after injury, their uninjured neighbors became more responsive, and this was associated with decreased mechanical activation thresholds in vivo and nociceptor sensitization to mechanical stimuli in vitro. We, for the first time, provide evidence that TRPA1 regulation is mediated by the IL-6 signal transducer gp130 in primary afferent nociceptors after nerve lesion using a transgenic mouse model. Overexpression of gp130 resulted in increased TRPA1 expression, and together with functional data, we propose that this upregulation in uninjured nociceptors is causally involved in the development and maintenance of neuropathic mechanical hypersensitivity.

Several seminal articles have linked important TRPA1 functions to the transition of mechanical hypersensitivity from an acute to a chronic condition.^62^ Despite the strengthening link between TRPA1 and neuropathic pain, it is still controversially discussed whether TRPA1 is indeed upregulated in primary nociceptive afferents in preclinical models of neuropathic pain.^11,50^

Mechanistically, hypersensitivity evoked by mechanical trauma is associated with intraneural and perineural monocyte and macrophage invasion and increased levels of oxidative stress by-products. AT2R in macrophages that infiltrate the site of injury trigger an intercellular redox communication and activation of the cell damage or pain-sensing ion channel TRPA1.^81^ Attenuation of monocyte or macrophage infiltration results in reduced pain-like behaviors, which are ablated by perineural administration of a TRPA1 antagonist, suggesting that pain-like behaviors may be entirely mediated by TRPA1.^88^ However, invading macrophages and monocytes can release a multitude of different bioactive compounds including immune mediators such as IL-6,^34^ and this is reflected by increasing levels of IL-6 but not gp130 in injured nerves and ganglia.^3,6,24,94^

Apart from invading macrophages releasing IL-6 in peripheral ganglia,^41,52^ neurons themselves are capable to synthesize IL-6 and contribute to increased IL-6 levels within neuropathic DRG.^86^ More recently, DRG satellite cells are emerging as an important IL-6 source after peripheral nerve injury.^25^ Another gp130 using cytokine, ciliary neurotrophic factor (CNTF), is highly expressed in Schwann cells and supports the neuroinflammatory response through the signal transducer and activator of transcription 3 (STAT3) and induction of IL-6 in sensory neurons. This Schwann cell–derived CNTF to neuronal STAT3 to neuronal IL-6 axis seems to mediate the onset and progression of the neuroinflammatory cascade resulting from nerve injury.^37^ Other central regulators, such as CCL2, affecting IL-6 levels further support the importance of IL-6/gp130 in the pathogenesis of neuropathic pain.^67^

Whereas cytokines, such as LIF or CNTF that use gp130 as their signal transducer subunit of their heteromeric receptors, are critically important for neuronal regenerative processes, IL-6 acting by gp130 homomeric receptors may have a broader function.^76^ Interleukin-6, like its related cytokines, promotes neuronal regeneration through gp130^72^, but also sensitizes nociceptors to mechanical stimuli,^7^ and IL-6 deficiency causes deficits both in regenerative and sensory properties of peripheral neurons.^98^ IL-6^−/−^ and SNS-gp130^−/−^ mice are protected from mechanical allodynia^73,74^ and a first hint towards an IL-6/gp130 regulated mechanosensitive ion channel and specifically TRPA1 emerged from our previous study using conditional gp130 depleted transgenic mouse model that exhibited reduced mechanonociception.^54^ Neuronally expressed gp130 is essential for the induction and maintenance of mechanical hypersensitivity experimentally induced by inflammation, tumor, or nerve injury.^73^ Based on these findings, we explored this link in the murine SNI model of neuropathic pain. Like IL6^−/−^ mice, mice with a selective depletion of gp130 in nociceptors were protected from SNI-induced mechanical hypersensitivity, suggesting that IL-6 acting on gp130 expressed by nociceptors is critically involved in a process involving the primary nociceptive afferent either by targeting an ion channel serving transduction or synaptic transmission mechanisms both involving TRPA1. This was supported by upregulated TRPA1 expression in cultured neurons after rescue or overexpression of gp130 with an adenoviral vector approach. Despite this observation, but similar to results in previous studies, TRPA1 mRNA upregulation was not detectable in DRG explants from SNI-treated mice. Although mRNA levels do not necessarily reflect protein levels, quantitative estimation of protein expression on a single cell level remains challenging. However, with a functional readout, using a well-accepted TRPA1 agonist, we demonstrated differential regulation of TRPA1 in injured vs uninjured neurons. This was further supported by the finding that upregulation of TRPA1 did not occur in uninjured sensory neurons obtained from SNS-gp130^−/−^ mice and signatures of mechanical hypersensitivity were largely absent in these mice.

Our current study provides a solution to the enigmatic and partially inconsistent reports of TRPA1 expression in neuropathic pain models where TRPA1 expression seems to depend on the impact of the injury on the respective neurons. Varying numbers of primary afferent neurons may be injured, for example, in the chronic constriction injury or the ligation models. These cannot easily be determined if the impact of the injury model affects the entire nerve, and this may even more apply to chemotherapy-induced neuropathic pain models.

Overall, the current findings support our idea that IL-6/gp130 signaling, likely by STAT3 as previously published,^54^ not only sets mechanosensitivity in healthy conditions but is also critically regulating TRPA1 in uninjured neurons, indirectly affected by neuropathic conditions. The differential upregulation of TRPA1 exclusively in uninjured but not in injured neurons provides important novel mechanistic insight into the critical role of TRPA1 in neuropathic pain pathogenesis and stresses the importance of this ion channel as a relevant drug target for neuropathic pain disorders.

## Conflict of interest statement

The authors have no conflicts of interest to declare.
